# Russian medical students in the first wave of the COVID-19 pandemic: emotional reactions and baseline beliefs

**DOI:** 10.1192/j.eurpsy.2022.1242

**Published:** 2022-09-01

**Authors:** Y. Aleksandrovich, D. Ivanov, I. Gorkovaya, V. Titova, V. Rozhdestvenskiy

**Affiliations:** Saint Petersburg State Pediatric Medical University, Department Of Psychosomatics And Psychotherapy, Saint Petersburg, Russian Federation

## Abstract

**Introduction:**

During the pandemic of new coronavirus infection, some medical students were actively recruited to work with infected patients, which could provoke depression, anxiety, and stress. The concept of baseline beliefs predicts characteristics of individuals’ experience of trauma.

**Objectives:**

The study aimed to determine depression, anxiety, and stress levels in medical students and examine their baseline beliefs, as well as the relationship between baseline beliefs and emotional reactions.

**Methods:**

Data were collected in the spring and summer of 2020 using a Google form that we developed. Thirty-seven medical students participated in the study. The WAS-37 questionnaire was used to examine baseline beliefs and the DASS-21 to measure depression, anxiety, and stress. Both questionnaires were adapted for use in Russia.

**Results:**

We found that 78 % of the respondents had no depression, 86 % had no manifestations of anxiety, and 83 % felt stress-free. The mean values on the “Benevolence in the World” scale (M = 32.3±8.0) were within the average normative values, those on the “Justice” scale (M = 19.8±5.0) were below them, and those on the “Self-Image” scale (M = 29.6±5.9), “Luck” (M = 32.5±6.9) and “Controlling Beliefs” (M = 27.3±4.1) were above the average normative values. We found only one statistically significant relationship between emotional reactions and baseline beliefs, a negative correlation between depression and luck (r_x_ = -0.360, p < 0.05).

**Conclusions:**

In pandemic medical students, beliefs about one’s luck were associated with lower levels of depression.

**Disclosure:**

No significant relationships.

